# ER stress sensor, glucose regulatory protein 78 (GRP78) regulates redox status in pancreatic cancer thereby maintaining “stemness”

**DOI:** 10.1038/s41419-019-1408-5

**Published:** 2019-02-12

**Authors:** Patricia Dauer, Nikita S. Sharma, Vineet K. Gupta, Brittany Durden, Roey Hadad, Santanu Banerjee, Vikas Dudeja, Ashok Saluja, Sulagna Banerjee

**Affiliations:** 1 Department of Pharmacology, University of Minnesota, Minneapolis, MN 55455 USA; 2 Department of Surgery, University of Miami, Miami, FL USA; 3 Sylvester Comprehensive Cancer Center, Miami, FL USA

## Abstract

Endoplasmic reticulum (ER) stress and the unfolded protein response (UPR) signaling have been shown to be dysregulated in multiple cancer types. Glucose regulatory protein 78 (GRP78), the master regulator of the UPR, plays a role in proliferation, invasion, and metastasis in cancer. Cancer stem cells (CSCs) make up a crucial component of the tumor heterogeneity in pancreatic cancer, as well as other cancers. “Stemness” in pancreatic cancer defines a population of cells within the tumor that have increased therapeutic resistance as well as survival advantage. In the current study, we investigated how GRP78 was responsible for maintaining “stemness” in pancreatic cancer thereby contributing to its aggressive biology. We determined that GRP78 downregulation decreased clonogenicity and self-renewal properties in pancreatic cancer cell lines in vitro. In vivo studies resulted in delayed tumor initiation frequency, as well as smaller tumor volume in the shGRP78 groups. Additionally, downregulation of GRP78 resulted in dysregulated fatty acid metabolism in pancreatic tumors as well as the cells. Further, our results showed that shGRP78 dysregulates multiple transcriptomic and proteomic pathways that involve DNA damage, oxidative stress, and cell death, that were reversed upon treatment with a ROS inhibitor, N-acetylcysteine. This study thus demonstrates for the first time that the heightened UPR in pancreatic cancer may be responsible for maintenance of the “stemness” properties in these cells that are attributed to aggressive properties like chemoresistance and metastasis.

## Introduction

Pancreatic cancer is a devastating disease with an estimate that 55,440 people will be diagnosed, of which 44,330 people will die in the United States in 2018 alone^1^. Compared with the 20 most prevalent cancers in the United States, pancreatic cancer is the only type that has a 5-year survival rate of <10% for all stages^1–9^. Thus, there is a need to understand the basic biology of pancreatic cancer with an emphasis on mechanisms for tumor recurrence in order to develop a viable therapeutic strategy.

One mechanism utilized during oncogenic reprogramming is the unfolded protein response (UPR). Apart from its usual role in regulating environment-induced stress, we and others have shown that UPR plays a vital role in conferring chemoresistance to cancer cells^10–12^. Endoplasmic reticulum (ER) stress and UPR signaling is dysregulated in many cancers^13–19^. Various physiological or xenobiotic pressures on the cell, like glucose deprivation, hypoxia, or chemotherapeutics induce ER stress, which activates an adaptive and survival response, namely the UPR, that helps the cell recover from stress. This seemingly innocuous homeostatic survival mechanism can be hijacked by cancer cells to aid in tumor growth, migration, transformation, and angiogenesis^13,14,20,21^. GRP78, the master regulator of the UPR, has been reported to be upregulated in multiple cancers^11,15,19,22–25^. In pancreatic cancer, it was recently reported that GRP78 is overexpressed^11,19,24^ and plays a role in proliferation, invasion, and metastasis^19,23^.

A small population of treatment-refractory cells within the tumor contribute to its aggressive phenotype by promoting metastasis and tumor recurrence^15,26–30^. This population, typically defined as “cancer stem cells (CSC)” makes up a crucial component of the tumor heterogeneity in pancreatic cancer, as well as other cancers^27,28,31–33^. In pancreatic cancer, we and others have shown that this aggressive population can be identified as a CD133+ population^27,33^. This population has increased resistance to therapy, showed increased metastatic potential and is also responsible for tumor recurrence and sustained tumorigenicity, and overexpressed GRP78^27,33^. Role of GRP78 in maintaining the survival of CSCs has not been studied extensively^34,35^. However, a recent study showed downregulation of inositol-requiring enzyme 1 alpha (IRE1α), one of three transmembrane sensors, resulted in a decrease of colonic CSC^36^. Additionally, a study using an inducible knockdown of GRP78 (*Grp78*^*f/f*^; *Mx1-Cre)* results in decreased hematopoietic stem cells, decreased lymphoid progenitors, decreased viability, increased UPR and cell death^37^. These studies suggest that GRP78 may play an important role in the survival of normal stem cells, but its role in cancer stem cells (CSCs) remains unclear.

UPR signaling is also important for maintaining low levels of reactive oxygen species (ROS) and transcriptionally regulating detoxifying enzymes^20,21,38,39^. Interestingly, CSCs typically undergo metabolic reprograming in order to maintain low levels of ROS^28,38^, since accumulation of ROS can lead to DNA damage and genomic instability^40–42^. It has also been reported that hematopoietic stem cell self-renewal capacity depends on inhibition of oxidative stress^43^. Furthermore, ER is a site for sterol and phospholipid synthesis. Maintenance of lipid homeostasis is important for normal cells, as well as cancer cells^44–47^. Rapidly proliferating cells demand more cholesterol and lipids, which are acquired exogenously or by upregulating lipogenesis pathways in a number of cancers^48–50^. Thus, disruption of ER stress regulation affects these processes as well.

In the current study, we defined the role of GRP78 in the biology of pancreatic CSC. We used a pancreatic cancer cell line stably expressing shGRP78 in order to study *how* this critical ER stress regulator was instrumental in determining the aggressive phenotype of pancreatic cancer. Our study showed downregulation of GRP78 not only disrupts multiple pathways that are key in proliferation, survival, fatty acid metabolism, and cell organization and biogenesis, but is also required for maintenance of redox balance and thus self-renewal properties in pancreatic cancer.

## Results

### Downregulation of GRP78 in pancreatic cancer cells affects metabolic pathways regulating survival

**Fig. 1:**
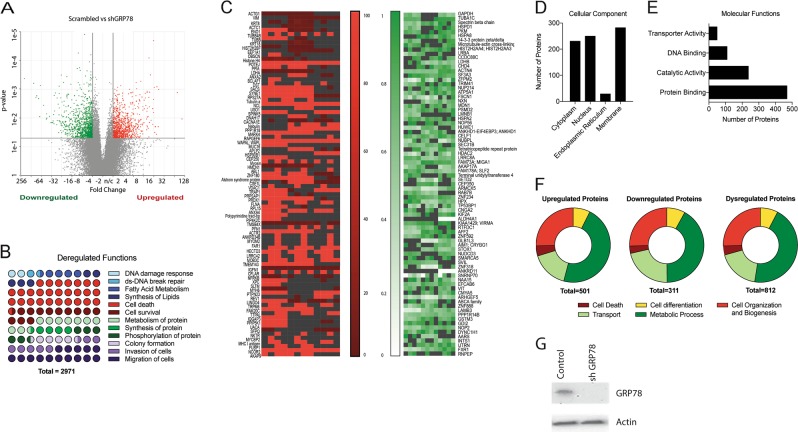
Downregulation of GRP78 in pancreatic cancer cells affects metabolic pathways regulating survival Transcriptomic analysis was performed on shGRP78 versus control cells (**a**, **b**). **a** Volcano plot depicting the significantly deregulated genes (red = upregulated; green = downregulated). **b** Dot plot depicting the percentage of significantly deregulated genes by function. iTRAQ proteomic analysis was performed on shGRP78 versus control cells (**c**–**f**). **c** Heatmaps showing upregulated (red) and downregulated (green) genes. Deregulated genes were sorted according to (**d**) cellular component (**e**) molecular function, and (**f**) biological function. **g** Downregulation of GRP78 in shGRP78 was confirmed by western blotting

In order to elucidate the role that GRP78 plays in addition to unfolded protein response and regulation of ER stress in pancreatic cancer, we constructed a sgGRP78 clonal cell line and conducted a transcriptomic analysis of our shGRP78 cells versus controls. We found a number of genes were significantly deregulated in our shGRP78 group (Fig. 1a). Supplementary Figure 1A lists the top 28 significantly deregulated canonical pathways. The top 25 dysregulated canonical pathways sorted by z-score are in Supplementary Figure 1B. Further, the top deregulated genes are organized according to function in Fig. 1b. There were 2971 genes dysregulated in shGRP78 cells compared to control, which are represented as 100 circles (100%). The number of colored circles is directly proportionate to the number of genes for that cellular function, in respect to the total number of genes.

To study the effect on the translatable protein components in response to GRP78 inhibition, we next conducted a quantitative iTRAQ proteomic analysis between shGRP78 and control cells. Data has been deposited in GEO GSE115849. Figure 1c depicts two heatmaps showing the upregulated (red) and downregulated (green) proteins of shGRP78 when compared to control cells. We found multiple canonical pathways dysregulated by GRP78 knockdown, including actin cytoskeleton signaling and protein ubiquitination. As seen with the transcriptomics study, we observed significantly deregulated NRF2-mediated oxidative stress response, glutathione depletion, and G2/M DNA damage checkpoint regulation (Table 1.1). Two hundred and sixty-eight proteins were involved in cell death and survival (*p* = 3.91E^−4^–2.38E^−13^), indicating a role of GRP78 in DNA damage and repair by maintaining the redox balance in the cells; 164 proteins involved in cellular assembly and organization (*p* = 3.53E^−4^–3.99E^−10^); 198 proteins involved in cellular function and maintenance (*p* = 5.35E^−4^–3.99E^−10^); 172 proteins involved in cell morphology (*p* = 5.35E^−4^–1.08E^−8^); and 161 proteins involved in cellular movement (*p* = 2.86E^−4^–2.79E^−7^) (Table 1.2).

**Table 1.1:** Significantly deregulated pathways in shGRP78 cells

Pathway	*p* Value
Actin cytoskeleton signaling	4.82E–08
Protein ubiquitination	3.32E–06
NRF2-mediated oxidative stress response	6.14E–04
Glutathione depletion—phase II reactions	2.77E–02
Cell death	2.89E–02
Cell cycle: G2/M damage checkpoint regulation	2.93E–02

**Table 1.2:** Significantly deregulated molecular functions in shGRP78 cells

Molecular function	*p* Value	No. of molecules
Cell death and survival	3.91E–04–2.38E–13	268
Cellular assembly and organization	3.53E–04–3.99E–10	164
Cellular function and maintenance	5.35E–04–3.99E–10	198
Cell morphology	5.35E–04–1.08E–08	172
Cellular movement	2.86E–04–2.79E–07	161

We categorized the observed dysregulated proteins according to their cellular component (Fig. 1d), molecular function (Fig. 1e), and biological function (Fig. 1f). The top significantly deregulated genes/gene clusters are identified in Supplementary Figure 1C, where the *q*-value ranged from 0.001 to 0.05. GRP78 inhibition in S2VP10 cells was confirmed by western blot (Fig. 1g).

### GRP78 knockdown induces proliferation defects in pancreatic cancer cells affecting their ability for self-renewal and invasion

**Fig. 2:**
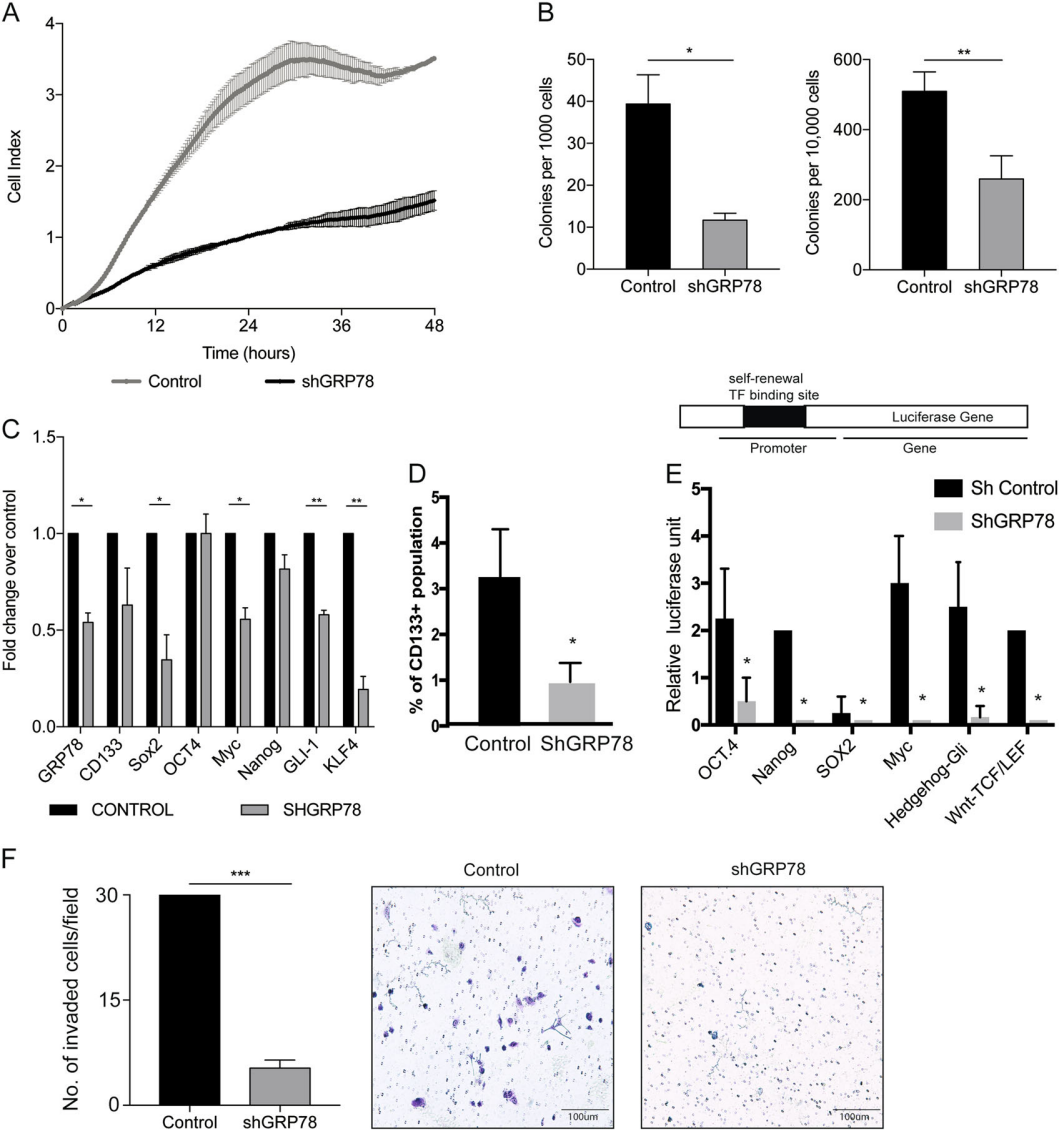
shGRP78 have decreased “stemness” and self-renewal phenotype. Control and shGRP78 cells were analyzed for **a** cell proliferation and **b** colony formation ability at limiting dilution, **c** mRNA expression of CD133 and self-renewal genes, **d** CD133+ population in control and shGRP78 cells was determined by flow cytometry, **e** a dual luciferase reporter assay on transcription factors (TF) regulating self-renewal in cells showed a decrease in the transcriptional activity of a number of TFs. **f** shGRP78 has lower invasiveness. Invasion images were taken with a ×4 objective and counted with Image J software

To study the phenotypic effect of GRP78 knockdown, we evaluated the proliferative ability of the cells. We observed that the shGRP78 cells had a slower proliferation rate compared to the sh-scrambled control (referred to as control) when analyzed real-time by electric cell-surface-impedance sensing or ECIS (Fig. 2a).

To study if the loss in proliferation was affecting the clonogenic ability of the pancreatic cancer cells, we next performed a limiting-dilution colony formation assay. After 6 days, the number of colonies formed in each of the groups was counted. shGRP78 cells had significantly fewer colonies formed than the control cells using both 10,000 and 1000 cells (Fig. 2b). No colonies were detected with shGRP78 in 100, 10, or 1 cell. This indicated that the low proliferative rate of the shGRP78 also affected their clonogenicity.

Clonogenicity is an indication of the ability of a tumor cell to self-renew and differentiate into a tumor in vivo and thus a measure of the “stemness”. To study if the decreased clonogenicity resulted in a reduced expression of self-renewal genes, we next studied the expression of self-renewal genes in shGRP78 cells. Our analysis showed that shGRP78 cells not only had decreased CD133 mRNA expression but also showed significant downregulation of other genes that are involved in self-renewal (Fig. 2c). This was consistent with our previously published data, in which CD133 + cells (having distinct survival advantage and show “stemlike” properties) isolated from pancreatic tumors showed an increase in GRP78 and other heat shock protein expression^33^ as well as with our transcriptomic profiling of these cells (Supplementary Figure 1B). Since our previously published data established CD133 + population as an indication of tumor initiating potential of pancreatic cancer, we next studied the effect on the population of CD133 + cells in shGRP78 cells. Our results showed that shGRP78 had significantly smaller number of CD133 + cells (Fig. 2d). Further, shGRP78 significantly decreased the transcriptional activity of multiple transcription factors involved in self-renewal pathways as observed by dual luciferase reporter assay (Fig. 2e)

Since our transcriptomics and proteomics analysis indicated that cell migration and invasion were affected upon knocking out GRP78, we next assessed the invasiveness of these cells and compared to control. Equal cell number of both control and shGRP78 cells were plated in the chamber and invaded colonies were measured after 24 h. Our results using a Boyden chamber assay with control or shGRP78 cells indicated that shGRP78 cells had a significantly reduced number of invaded cells compared to the control cells (Fig. 2f).

### Tumor initiation and progression are significantly reduced in GRP78 knockdown mice

**Fig. 3:**
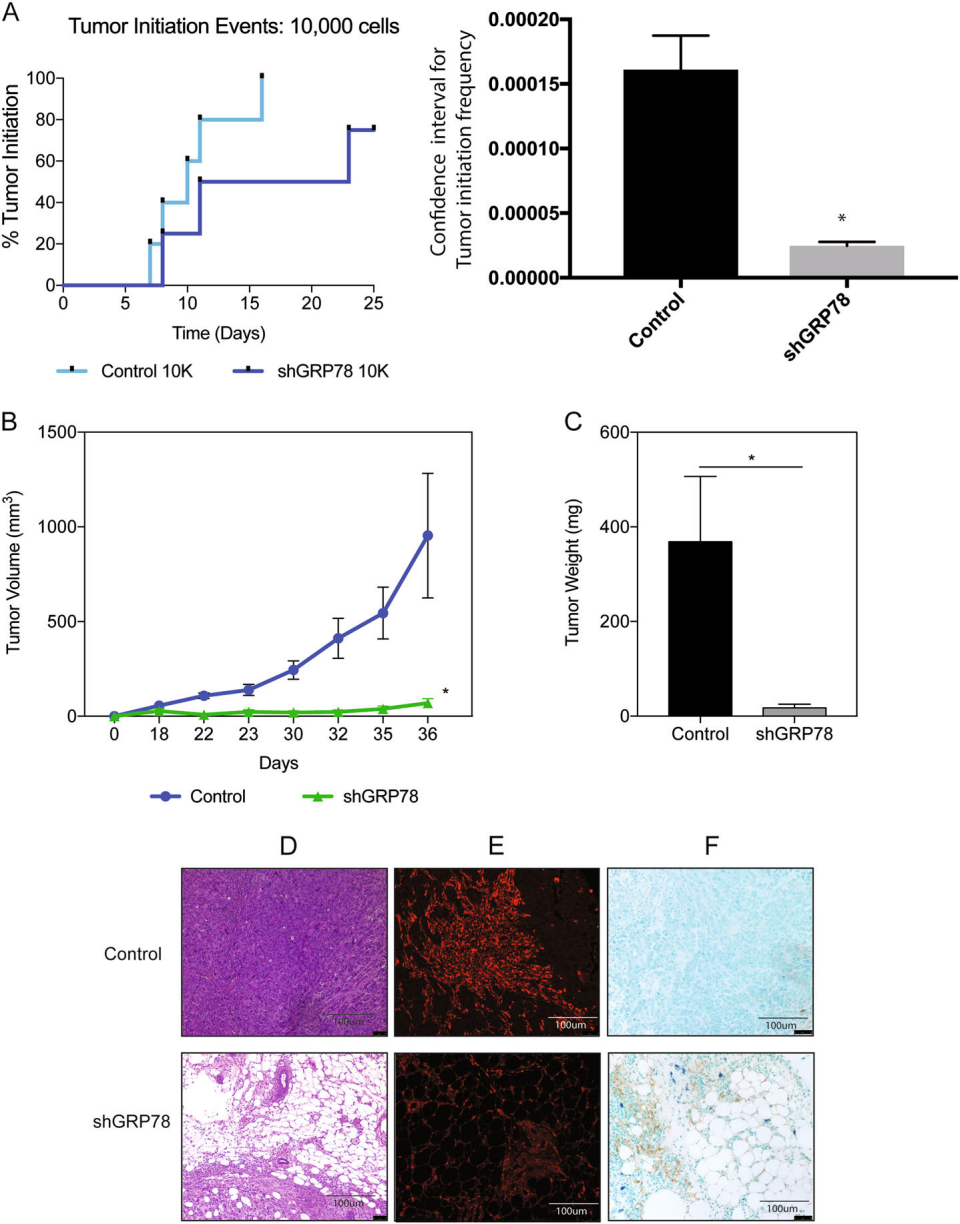
Tumor initiation and progression are significantly reduced in GRP78 knockdown mice. Athymic nude mice were injected with 10,000 cells. **a** Tumor initiation was monitored daily for 25 days. Tumor initiation frequency was determined at 16 days when all the animals in control group developed tumors. **b** shGRP78 cells showed delayed tumor progression along with delayed tumor initiation. **c** Tumor weights were noted at endpoint. **d** H&E of control and shGRP78 tumors. Control and shGRP78 tissues stained with **e** GRP78 and **f** TUNEL. Images were acquired with a ×20 objective

Since we observed a distinct difference in clonogenicity in shGRP78 cells, we next evaluated the tumor initiation frequency in vivo. To determine this, we injected pancreatic cancer cells in limiting dilutions subcutaneously into athymic nude mice. Animals were monitored for tumor initiation daily. Tumor initiation frequencies were calculated according to extreme limiting dilution assay or ELDA (http://bioinf.wehi.edu.au/software/elda/). Our results showed that while 1/6214 control cells have probability to initiate a tumor, whereas only 1/45217 shGRP78 cells has the probability of initiating a tumor, indicating a decrease in tumor initiation potential (Fig. 3a). In addition to delayed tumor initiation, shGRP78 mice (both groups) had significantly smaller tumor volumes (Fig. 3b, Supplementary Figure 2B) and significantly less tumor weight in the shGRP78-10,000 cell group (Fig. 3c) compared to control mice after 4-week post-implantation. Even though the control-100,000 cells group had significantly greater tumor volumes than the corresponding shGRP78 group, many of the S2-VP10 tumors result in ascites and/or ulceration, thereby reducing the overall tumor weight upon necropsy (Supplementary Figure 2C).

Examination of the H&E from the control and shGRP78 mice showed pronounced accumulation of lipids in and around the tissues of the shGRP78 mice (Fig. 3d, Supplementary Figure 2D). The formaldehyde fixed tissue sections stained with GRP78 confirmed reduction of GRP78 in vivo (Fig. 3e, Supplementary Figure 2E). We also observed more apoptotic cells in the shGRP78 mice, when stained with TUNEL (Fig. 3f, Supplementary Figure 2F).

### Downregulation of GRP78 leads to deregulated lipid metabolism

**Fig. 4:**
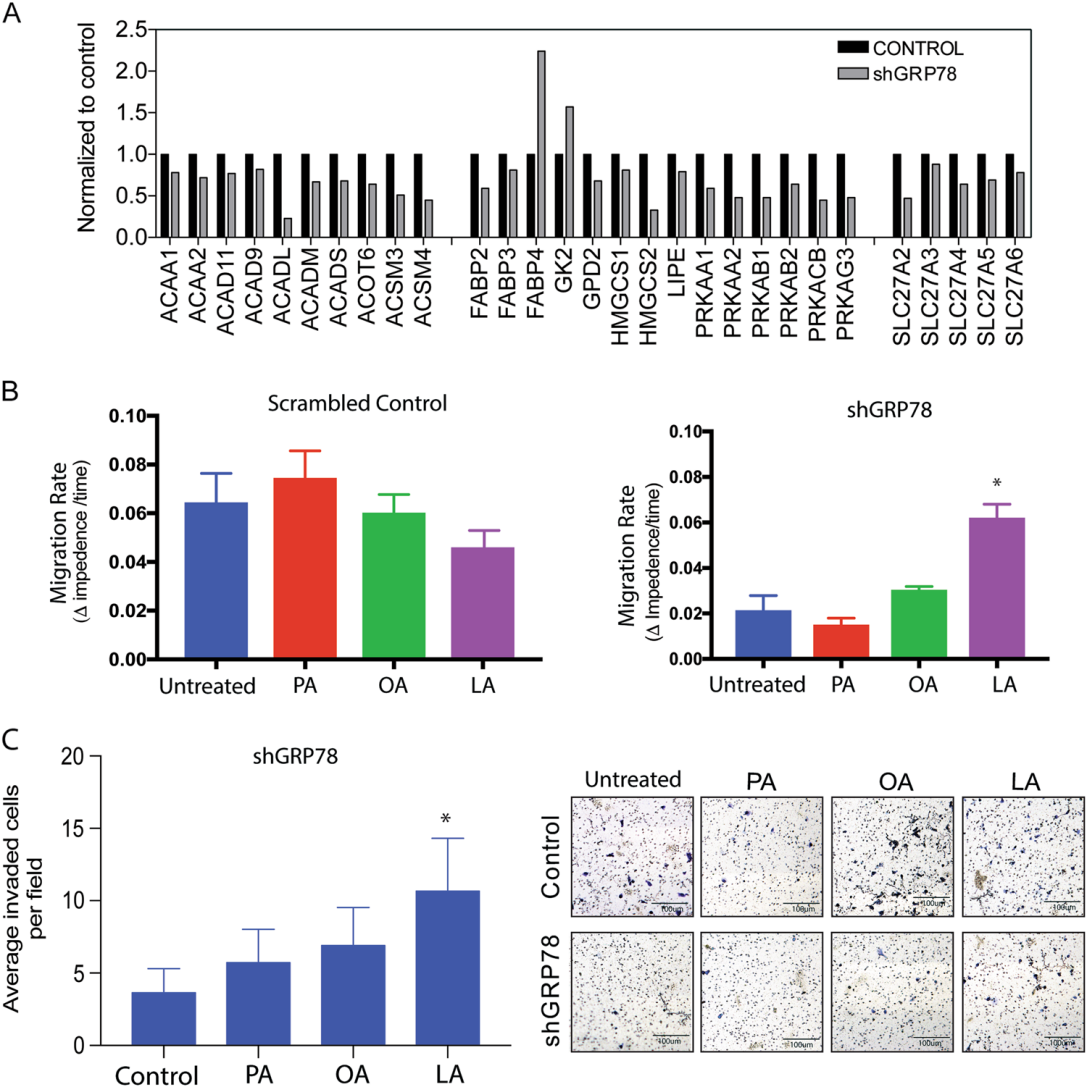
Downregulation of GRP78 results in deregulated fatty acid metabolism. **a** shGRP78 has dysregulated fatty acid metabolism genes in vitro. **b** The addition of fatty increased cell migration in 30 h in shGRP78 while it had no significant effect on control cells. **c** Fatty acid supplementation increases cell invasion using a Boyden chamber assay. Invasion images were taken with ×5 objective and counted using Image J software

Our transcriptomic analysis showed that a large number of proteins involved in fatty acid synthesis and lipid metabolism was deregulated. Further, tumors from shGRP78 cells showed pronounced lipid accumulation. To study this further, we used a qPCR array for fatty acid metabolism. Our results showed that a large number of fatty acid metabolism genes were being downregulated in shGRP78 cells (Fig. 4a). These in vitro results were consistent with mRNA from our subcutaneous tumors using the same fatty acid metabolism qPCR array (Supplementary Figure 3).

Lipid metabolism in cancer has been implicated to play a role in cell proliferation. Since shGRP78 cells had dysregulated fatty acid metabolism as well as decreased proliferation, we next supplemented pancreatic cancer cells with saturated (SFA) and unsaturated fatty acids (UFA), to see if lack of proliferation in shGRP78 cells could be rescued. It has been reported that SFAs induce ER stress, but UFAs can inhibit ER stress. We treated control and shGRP78 cells with palmitic acid (SFA), linoleic acid (UFA), or oleic acid (UFA) for 24 h and determined cell viability, proliferation, and invasion ability. Supplementation of fatty acids did not significantly affect cell viability (data not shown), or proliferation (Supplementary Figure 3B). However, supplementation of Linoleic acid rescued the migration of shGRP78 cells (Fig. 4b) as well as increased the invasiveness of cells in vitro (Fig. 4c). The effect of palmitic acid and Oleic acid on invasion and migration was modest in shGRP78 cells. Fatty acid supplementation did not affect invasiveness of the control cells (as seen in the image in Fig. 4c).

### GRP78 inhibition leads to deregulated redox balance in the cells resulting in increased lipid-peroxidation and ROS accumulation

**Fig. 5:**
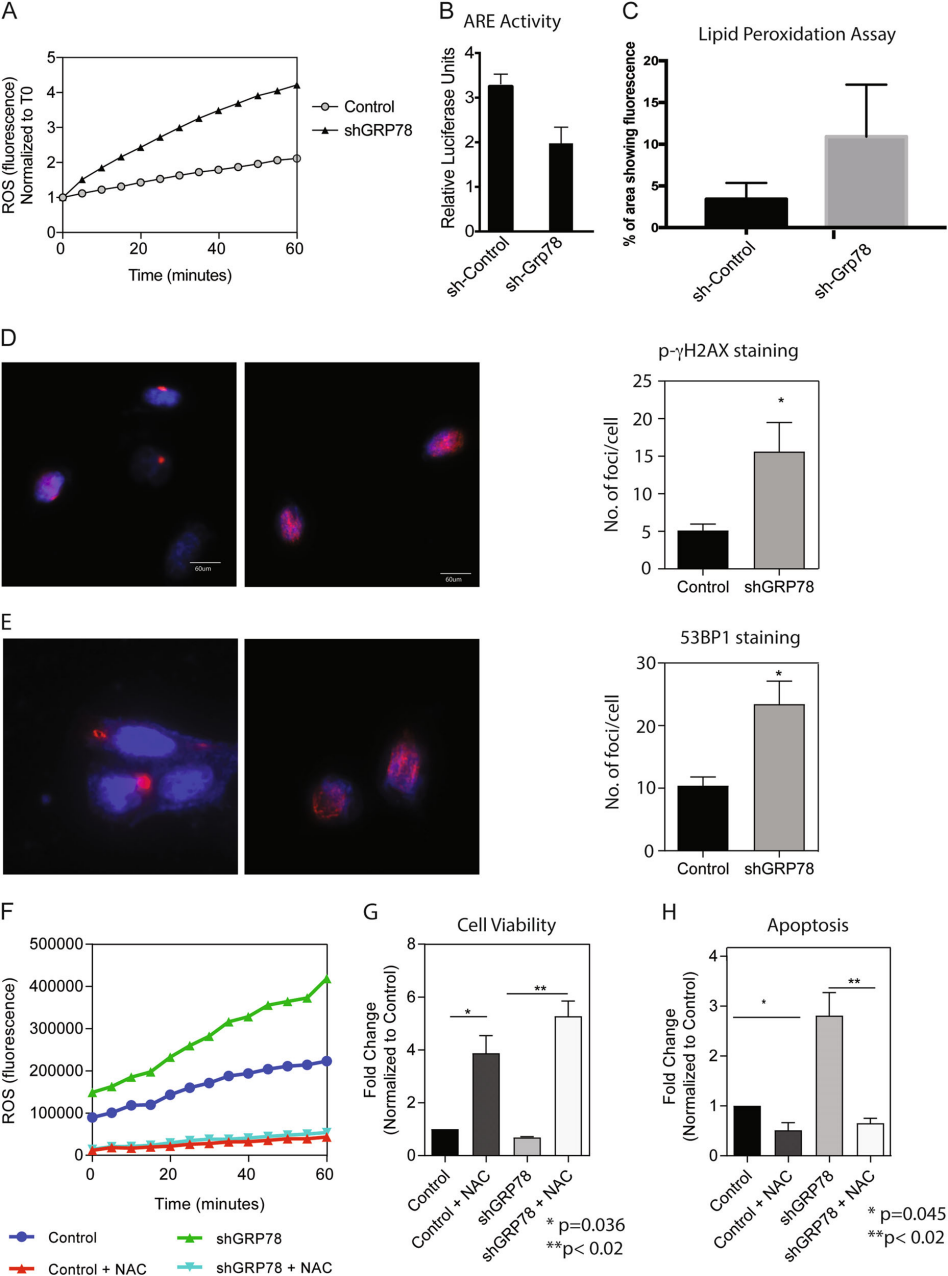
GRP78 knockdown leads to deregulated redox balance and DNA damage. **a** ROS fluorescence of control and shGRP78 measured at 5-min intervals over 60 min. Inhibition of GRP78 resulted in a decreased NRF2 activity as shown by the ARE dual luciferase assay (**b**), shGRP78 tumors showed an increase in lipid-peroxidation compared to the control (**c**). After 48 h of plating, control and shGRP78 cells were stained with **d** γ-H2AX and **e** 53BP1 to measure DNA damage. Cells were quantitated and expressed as mean intensity/cells per field. Control and shGRP78 cells were treated +/−10 mM N-acetyl-cysteine (NAC) for 24 h. **f** ROS fluorescence was measured at 5-min intervals over 60 min, **g** cell viability after 24 h NAC treatment, and **h** caspase 3/7-mediated activity after 24 h of NAC treatment

Our recently published results show that GRP78 is instrumental in maintaining NRF2 activity in pancreatic cancer cells, and this contributes to the chemoresistance in this cancer by regulating the oxidative stress response in the cancer cells^10^. Our earlier studies showed that the treatment-refractory CD133 + pancreatic cells maintain a low ROS status in order to maintain a survival advantage^28^. In this study, we observed that shGRP78 cells have a higher ROS baseline compared to control cells (Fig. 5a). Consistent with this, these cells had less NRF2 transcriptional activity as seen by anti-oxidant response element or ARE dual luciferase reporter assay (Fig. 5b) and an increased lipid-peroxidation (Fig. 5c),

To study if increased ROS in shGRP78 cells also corresponded with an increased DNA damage potential, we next investigated the baseline level of DNA damage by staining control and shGRP78 cells with phospho-γ-H2AX, a marker for double stranded breaks (Fig. 5d). Cells were stained 48 h after plating and the number of foci/cell staining positive for phospho-γ-H2AX was counted in both control as well as shGRP78 cells to represent the data quantitatively. Our study showed that shGRP78 had a significantly high number of foci staining positive for the phospho-γ-H2AX indicating that these cells had increased DNA damage. We further confirmed these findings by staining for 53BP1, another marker of DNA damage (Fig. 5e). As observed with phospho-γ-H2AX, 53BP1 also showed that shGRP78 had increased DNA damage.

Since low basal ROS production and accumulation is a protective mechanism that prevents aggressive cancer cells from oxidative stress induced cell death and apoptosis, we next evaluated if inhibiting ROS production with N-acetyl-cysteine (NAC) in shGRP78 would result in a rescue in cell viability and apoptosis, and thus help in maintenance of “stemness” that contributes to the resilience of cancer cells. We found that treatment of 10 mM NAC for 24 h lowered ROS levels in shGRP78 cells in comparison to control cells (Fig. 5f). We also found that treating control and shGRP78 cells with 10 mM NAC for 24 h rescued cell viability (3.9-fold and 5.3-fold over untreated control cells, respectively) (Fig. 5g).

To study if the DNA damage observed in Fig. 5d, e was due to increased ROS in the shGRP78 cells, we performed a comet assay on control and shGRP78 cells in the presence of N-acetyl-cysteine. Our study showed that in the control cells there was very little damage (as shown by most cells showing a C0 or a C1 extent of damage. When treated with NAC, there was no significant changes observed (Supplementary Figure 4A). Similarly, shGRP78 cells showed more cells with C1-C3 extent damage which were rescued when treated with NAC. This indicated that increased ROS was contributing to DNA damage in shGRP78 cells. However, the rescue observed with NAC, while significant in C1 and C2 damage was not overwhelming in C3 damage (Supplementary Figure 4B). This indicated that while ROS was contributing to DNA damage, there were probably other factors that may be involved in this as well.

Further, untreated shGRP78 cells had a 2.8-fold increase in caspase 3/7-mediated cell death when compared to untreated control cells. Treatment with 10 mM NAC for 24 h rescued shGRP78 cells from caspase 3/7-mediated apoptosis (Fig. 5h). Together, this suggested that GRP78 in addition to maintaining ER homeostasis played a vital role in protecting the cancer cells from oxidative stress induced cellular damage and cell death.

## Discussion

It is well established that tumors use UPR and ER stress as a survival pathway. Previously published studies from our laboratory has shown that increased UPR in pancreatic cancer cells contribute to therapeutic resistance^10^. Further, aggressive pancreatic cancer population represented by CD133 + cells also show an increased expression of GRP78 one of the critical ER stress regulators^33^. Upon activation of the pro-apoptotic branches of the UPR, cancer cells with mutations in the apoptotic machinery are selected in order to evade cell death. ER stress also induces pro-survival pathway NF-κB and inhibits p53-dependent apoptotic signals. In the event of a pro-survival UPR activation, ER stress can promote aggressive growth of these cancer cells by enhancing their angiogenesis^14^. In vitro studies show that inhibiting GRP78 in immortalized endothelial cells decreases proliferation, survival, and migration^23^. Additionally, GRP78 was critical for survival as GRP78^−/−^ mouse models were embryonic lethal^47,51^. Studies have also shown that knocking down GRP78 decreases invasion and metastatic growth in vivo^23^.

In this study, we showed that knocking down GRP78 dramatically altered genes involved in DNA damage, oxidative stress signaling, cell proliferation and cell death (Fig. 1). GRP78 is known to play an important role in maintaining low levels of oxidative stress and DNA damage^20,21,38,39^. DSBs can occur from DNA replication stress, which can activate DNA repair genes, leading to cell cycle arrest, senescence, or cell death^41,42^. Additionally, knocking down GRP78 results in an increased ROS production that led to increased lipid-peroxidation in the tissue samples lacking GRP78 (Fig. 5c). This was likely to be responsible for more DNA DSBs, as indicated by phospho-γ-H2AX and 53BP1 (Fig. 5d, e). However, the evidence for more DNA damage in the shGRP78 cells may also provide insight into how the shGRP78 cells have a slower proliferation rate and result in less tumorigenicity (Figs. 2, 3).

While there has not been a lot of studies exploring role of ER stress and self-renewal in cancer, in head and neck cancer, inhibiting GRP78 decreased tumor initiation^22^. In this study, we show for the first time, a connection with the UPR and self-renewal in pancreatic cancer (Figs. 2, 3). Recent literature, particularly those highlighting the importance of the niche in selection for CSC has redefined the concept of “stemness” in cancer. This population in a tumor need not necessarily be the cell of origin in a tumor, but rather a population of tumor that has adapted to the inhospitable tumor microenvironment and oncogenic stress pathways in order to be a treatment-refractory population. This population is thus resistant to most chemotherapy by upregulation of ABC transporters and detoxifying enzyme activity like Aldehyde dehydrogenases. This is in accordance with our previous observation that pancreatic “CSCs”, expressing CD133 on their surface, typically maintain a low redox status. This prevents ROS accumulation in these cells and confers to their chemoresistant phenotype^28^. Our study has also shown that upsetting this redox status to elevate ROS leads to apoptotic cell death in this population^28^, indicating that low redox status offers a survival advantage in these cells. Interestingly, previous studies from our lab has also shown these treatment-refractory CD133 + pancreatic cells have also shown that these cells typically have a high GRP78 expression, which presumably contributes to their increased survival advantage^33^. These results indicate that increased GRP78 in this population is responsible for the chemoresistant property of the CD133 + pancreatic cells by maintaining a redox balance in these cells.

Inhibiting GRP78 in pancreatic cancer seems to have a profound effect on fatty acid metabolism as well. ER stress can lead to lipogenesis and altered metabolism, whereas lipids and aberrant metabolism can lead to ER stress^45^. Cancer cells require more lipid production for survival and proliferation^46,47^. Recent studies by Cook et al. showed that when GRP78 was inhibited in breast cancer, there was reduced fatty acid oxidation and increased cellular fatty acids^50^. Further, fatty acid synthase (FASN) is a multi-enzyme protein that catalyzes fatty acid synthesis. FASN expression correlates with cancer progression, poor therapeutic response to gemcitabine, and survival^46,52^. In addition, inhibiting FASN with orlistat has shown to decrease stemness in pancreatic cancer^52^. Role of lipids in cancer stem cell maintenance as well as a regulator of their phenotype is being acknowledged in recent studies^53–55^. Thus, dysregulation of lipid metabolism upon inhibition of GRP78 is likely to have an effect on the self-renewal in this disease.

It has been reported that multiple cancer cell lines require unsaturated lipids^46^. Under hypoxia, enzymatic reactions for de novo lipid synthesis are inactive, because they require oxygen^46^. As a result, cancer cells require exogenous unsaturated lipids, which can be acquired by desaturation of de novo fatty acids or by fatty acid uptake. Cells normally satisfy their fatty acid requirements from blood when triglycerides and low-density lipoproteins are hydrolyzed by lipoprotein lipase^46^. However, cancer cells are rapidly proliferating, and as a result, de novo lipid biogenesis provides most necessary lipids^47^. As seen in the current study, inhibition of GRP78 has a profound effect on the lipid metabolism pathway in the pancreatic cancer cells (Fig. 5). Inherent properties that are associated with an aggressive tumor (increased invasion, migration) are dependent on the lipid metabolism for reorganization of membrane architecture (that is critical for EMT and thus metastasis). This can be seen from out data that when linoleic acid is supplemented to shGRP78, there is a substantial rescue in migration (Fig. 5b) along with invasion (Fig. 5c) of the cells. Inhibition of GRP78 has been shown to downregulate sterol regulatory element-binding protein (SREBP) transcription factors in breast cancer^46,50^.

## Conclusion

Our current study demonstrates GRP78 is required for maintenance of “stemness” in pancreatic cancer and downregulating it interferes with cellular metabolism that contributes to survival advantage of treatment-refractory CSC population.

## Methods

### Cell culture and treatments

S2-VP10 (a gift from Dr. Masato Yamamoto’s lab, University of Minnesota) cells were cultured in RPMI 1640 containing 10% fetal bovine serum and 1% penicillin/streptomycin. Cells were treated with 1 µM palmitic acid (Sigma), 10 µM linoleic acid (Sigma), or 400 µM oleic acid (Sigma) in 1% BSA. Cells were treated with 10 mM N-acetyl-cysteine (Sigma).

### Plasmid

Four pGFP-C-shLenti-HSPA5 plasmids and one pGFP-C-shLenti-scrambled target were acquired from Origene (Cat # TL312303).

### Generation of stable cell line

S2-VP10 cells were transfected with one of four sh-GRP78 plasmids or scrambled sh plasmid (Origene, TL312303). In total, 1 μg/mL puromycin selection was applied after 48 h post transfection. After verifying GFP expression, cells were collected and brought to a Flow Cytometry Core Facility. Cells were gated by GFP-positive expression, and sorted single cells into each well of a 96-well plate. Clones were propagated and maintained in RPMI with 1 μg/mL puromycin.

### Migration assay

Migration assay was conducted by electric cell-substrate impedance sensing (ECIS) (Applied Biophysics). In ECIS, the cells are grown on the surface of small and planar gold-film electrodes and the AC impedance of the cell-covered electrode is measured continuously at a frequency of 64 kHz. In total, 4 × 10^4^ pancreatic cancer cells (Control, shGRP78) were seeded into an 8E1W ECIS array (Applied Biophysics) containing a 250 μM electrode. Upon confluence a high-field current with frequency 48 kHz and amplitude 5 was applied for 10 s killing cells overgrowing the electrode, creating “wounds” in the wells devoid of cells. The migration was compared between each cell line at the end of each experiment, which was presented as a measure of changing impedance over time after normalizing to the time for wounding. Migration potential of the cells determined according to the formula: impedence at [*T*_(final)_ − impedence at *T*_(initial)_]/total time of study, where *T* was time in hour.

### Transcriptomic analysis

Aliquots of RNA were derived from the qRT-PCR samples. Three control replicates and three shGRP78 replicates were analyzed. The RNA was quality tested using a Bioanalyzer 2100 (Agilent Technologies). cDNA was created by reverse transcription of oligo-dT purified polyadenylated RNA and fragmented, blunt-ended, and then ligated to barcoded adaptors. Then, the library was size selected, and the selection process was validated and quantified by capillary electrophoresis and qPCR, respectively. Samples were loaded on the HiSeq 2500 (Illumina Inc.) to generate around 25 million paired-end 50 bp reads for each sample. Quality control was conducted by FastQC 0.11.2 according to http://www.bioinformatics.babraham.ac.uk/projects/fastqc. The mean inner distance was established using the insertion size metrics feature of Picard-tools. The resulting TopHat data served as input to other Cufflinks tools 2.2.0. Transcripts were also assembled using Cufflinks, with stipulating the reference transcriptome *Homo sapiens*. All cufflinks assemblies were merged with Cuffmerge. Differentially expressed genes (DEGs) were calculated with Cuffdiff and presented the data in terms of fragments per kilobase of transcript per million mapped reads (FPKMs). Visualization of data from Cuffdiff outputs was used CummeRbund v2.0.0^56^. Ingenuity Pathway Analysis (Qiagen) was used for pathway enrichment analysis.

### iTRAQ proteomic quantification

Four control cell pellets and four shGRP78 pellets were prepared for iTRAQ MS/MS. To each cell pellet (each containing ~ 50 μg protein) 30 μL of dissolution buffer (0.5 M triethylammoniumbicarbonate (TEAB), pH 8.5) was added. Samples were denatured with 2% SDS, and reduced with 100 mM tris-(2-carboxyethyl) phosphine (TCEP). Samples were incubated for 1 h at 60 °C, and then 1 μL of 84 mM iodoacetamide solution was added, and incubated at 30 °C in the dark. Promega Sequencing Grade trypsin was added to each sample, and then incubated at 37 °C overnight. 8Plex iTRAQ reagents were prepared the following day by adding 50 μL isopropanol to each reagent. Each reagent was then transferred to one sample each. Sample-reagent mixtures were incubated for 2 h at room temperature, and then the reactions were quenched with water, and combined into one tube. Sample was then vacuum dried, and washed three times with water.

### Gene expression analyses

#### RT-PCR

RNA was isolated from the cells according to manufacturer’s instructions using Trizol (Invitrogen). Total RNA (2 μg) was used to make cDNA and perform real-time PCR using the Quantitect SyBr green PCR kit (Qiagen) according to the manufacturer’s instructions using Roche 480 real-time PCR system. All data were normalized to the housekeeping gene 18S (18s Quantitect Primer Assay; Qiagen). Primers for HSPA5 (GRP78), CD133, SOX2, OCT4, NANOG, GLI-1, and Myc were acquired from Qiagen.

Fatty acid metabolism array (Qiagen) was used to study expression level of genes involved in fatty acid metabolism as described by manufacturer’s instructions.

### ECIS

Proliferation was measured by electric cell-substrate impedance sensing (ECIS). Control and shGRP78 cells were seeded in 8W10E + PET array with or without fatty acid supplementation (see the ‘Cell culture and treatments' section). Impedance (Z), capacitance (C), and resistance (R) were monitored for 48 h by an ECIS Model Zϴ instrument (Applied BioPhysics Inc.) and normalized to Z0.

### Colony formation assay

One million control and shGRP78 cells were prepared in 1 mL PBS. Cells were then serially diluted, and 100 μL of the diluted cells were added to the cell resuspension solution (R&D Systems). Control and shGRP78 cells were both plated in multiple dilutions ranging from 100,000 cells to 1 cell. This cell/resuspension mixture was added to 1.8 mL of methyl cellulose media, and plated in a six-well plate with a blunt cannula. After 6 days, the number of colonies were counted for each dilution.

### Reactive oxygen species assay

shGRP78 and control cells were seeded in black, clear bottom 96-well plates. Media was removed and replaced with 5 μM H2DCFDA/phenol-free media for 1 h at 37 °C. Cells were washed with PBS, and phenol-free media was replaced. ROS was measured at 5-min intervals for 1 h, with 492/517 ex/em filters. Results were expressed as ROS fluorescence per viability using a WST-8 cell cytotoxicity assay (Dojindo).

### Cell viability assay

shGRP78 and control cells were seeded in a 96-well plate (7000 cells/well) and allowed to adhere for 24 h. Cell viability assay was performed using a WST-8 based cell cytotoxicity assay per the manufacturer’s protocol (Dojindo) and expressed after normalizing to untreated cells.

### Caspase 3/7 activity

shGRP78 and control cells were seeded in a white 96-well plate (10,000 cells/well) and allowed to adhere for 2 h. Cells were then treated with 10 mM N-acetyl-cysteine for 24 h. Caspase 3/7 analysis was performed per manufacturer’s protocol (Promega). Caspase results were normalized to cell viability, which was plated in tandem.

### Comet assay for DNA damage

Comet assay was performed on the control and shGRP78 cells before and after treating them with 10 mM N-acetylcysteine for 24 h. Assay was done using the CometAssay Kit from Trevigen according to manufacturer’s protocol. For extent of DNA damage the controls provided by the kit was used. In this kit C0 corresponded to no DNA damage while C4 corresponded to maximum DNA damage. The microscopy images were quantitated and represented as no. of cells/field, showing the extent of DNA damage C0–C4.

### Boyden chamber invasion assay

Boyden chamber invasion inserts (Corning biocoat) were rehydrated for at least 1 h in serum-free medium at 37 °C. Cells were plated into the inserts in serum-free medium. The wells contained the attractant: 10% fetal bovine serum containing medium. After 24 h, the top of the insert was washed with PBS, and inserts were fixed in methanol and stained with crystal violet. Membranes were analyzed using a light microscope. Cells were counted using Image J software.

### Lipid-peroxidation assay

Lipid-peroxidation was performed on tumor tissues (from Control and shGRP78 cells) using the ImageIT Lipid-peroxidation kit (Thermo-Fisher) following manufacturer’s protocol.

### In vivo study

10,000 cells (control or shGRP78), and 100,000 cells (control or shGRP78) were resuspended in 50:50 PBS:matrigel mixture, and injected into the right flanks of athymic nude mice (5 mice per group). Tumor formation was monitored daily, and tumor volume was measured using a digital caliper. When the endpoint of the experiment was reached, mice were sacrificed, and tissues were resected and sent to pathology for embedding and preparation of H&E and unstained slides. All procedures were approved by the University of Miami Institutional Animal Care and Use Committee (IACUC).

### Dual luciferase reporter assay

Control and shGRP78 cells were plated at a density of 5 × 10^5^ cells/well in a 24-well plate. Dual luciferase assay was performed using the Stem Cell Reporter Array kit (Qiagen) according to manufacturer’s instruction.

### Determination of CD133+ population

Control and shGRP78 cells were stained with anti-CD133-PE antibody (Miltenyi Biotech) for 20 min on ice. Cells were washed once in PBS, following which CD133 + population was detected by flow cytometry on a BD FACS Canto II.

### Immunohistochemistry

Paraffin-embedded tissues were deparaffinized in xylene and then rehydrated in graded ethanol. TUNEL staining was completed per manufacturer’s instructions (Abcam, Cat # ab206386). Briefly, slides were permeabilized with a proteinase K solution, and covered with TdT equilibration buffer. Tissues were then labeled with a TdT labeling reaction mixture for 1.5 h, and then a conjugate solution for 30 min. DAB solution was added for 15 min, and then slides were washed with water. Slides were counterstained with methyl green and mounted with permount. Images were obtained on a Leica DM6B with a ×20 objective.

### Immunofluorescence

Slides were deparaffinized in xylene and rehydrated through graded ethanol solutions. Antigen retrieval was completed in a steamer using Reveal Decloaker (Biocare Medical) and blocked with Dako serum block. Primary antibody for GRP78 (Cell Signaling) was diluted 1:200 in Sniper (Biocare Medical). Alexa 555-conjugated donkey anti-rabbit IgG (Molecular Probes) secondary antibody was diluted 1:1000. The slides were mounted using Prolong Gold anti-fade with 4′,6-diamidino-2-phenylindole (Molecular Probes). Immunofluorescence images were obtained on a Leica DM6B with a ×20 objective.

### Statistical analysis

Values are expressed as the mean +/− SEM. All in vitro experiments were performed at least three times. The significance between any two samples was analyzed by *t* test, values of *p* < 0.05 were considered statistically significant.

## Supplementary information

Supplementary Figure Legend

Supplementary Table

Supplementary Figure 1

Supplementary Figure 2

Supplementary Figure 3

Supplementary Figure 4
