# Supplementary material for: ER stress sensor, glucose regulatory protein 78 (GRP78) regulates redox status in pancreatic cancer thereby maintaining “stemness”
Source: Cell Death Dis. 2019 Feb 12;10(2):132. doi: 10.1038/s41419-019-1408-5 (PMC6372649; doi:10.1038/s41419-019-1408-5)
Supplement: Supplementary file 1 — Supplementary Figure Legend [file 41419_2019_1408_MOESM1_ESM.docx]

**Supplementary Figure Legends**

**Supplementary Figure 1: Significantly deregulated canonical pathways**

Transcriptomic analysis was performed on shGRP78 versus control cells (A-B). (A) The top 28 canonical pathways significantly deregulated. (B) The top 25 deregulated pathways sorted by z-score. iTRAQ proteomic analysis was performed on shGRP78 versus control cells, and (C) depicts significantly deregulated genes/gene clusters.

**Supplementary Figure 2: GRP78 knockdown results in delayed tumor initiation and smaller tumors**

Athymic nude mice were injected with 100,000 cells. (A) Tumor initiation was monitored daily for 25 days. (B) Tumor volume was measured weekly for 36 days. (C) Tumor weights were noted at endpoint. Quantitation of infiltrated adipocytes (D),GRP78 staining (E) and TUNEL staining (F) in the tumors.

**Supplementary Figure 3: Downregulation of GRP78 results in deregulated fatty acid metabolism**

shGRP78 has dysregulated fatty acid metabolism genes *in vivo (A)*. Supplementation of shGRP78 cells with palmitic acid, oleic acid or linoleic acid did not alter their proliferation (B).

**Supplementary Figure 4: Inhibition of GRP78 result in DNA damage due to ROS which is rescued by N-acetylcysteine**

Comet Assay showed shGRP78 cells had more DNA damage (more cells/field showing C1-C3 DNA damage compared to the control cells. This was partially rescued upon treatment with N-acetyl cysteine, a ROS inhibitor. Representative images are shown.
