# Supplementary material for: ER stress sensor, glucose regulatory protein 78 (GRP78) regulates redox status in pancreatic cancer thereby maintaining “stemness”
Source: Cell Death Dis. 2019 Feb 12;10(2):132. doi: 10.1038/s41419-019-1408-5 (PMC6372649; doi:10.1038/s41419-019-1408-5)
Supplement: Supplementary file 2 — Supplementary Table [file 41419_2019_1408_MOESM2_ESM.docx]

Supplementary Table

UPREGULATED GENES

| ACTG1 | VDAC1 | | | SFPQ | | HNRNPUL1 | | ZFP106; ZNF106 |  |  |
| --- | --- | --- | --- | --- | --- | --- | --- | --- | --- | --- |
| VIM | TRAP1 | | | NKTR | | API5 | | BPTF |  |  |
| KRT8 | PRPSAP1 | | | MYCBP2 | | HNRNPK | | BAZ2A |  |  |
| ACTC1 | PRDX1 | | | HLA-B | | PRPS2 | | POLR3H |  |  |
| ENO1 | PRDX1 | | | FUBP1 | | PGD | | KCNJ8 |  |  |
| TUBB4B | FLNA | | | NCOR2 | | CDC34 | | CSDE1 |  |  |
| TUBB | RPL15 | | | AKAP9 | | BZW1 | | SNX33 |  |  |
| KRT18 | ANXA4 | | | TTC27 | | EIF3CL | | NTRK3 |  |  |
| HIST2H2BF | PTBP1 | | | ZBTB4 | | NPM1 | | GTPBP10 |  |  |
| EEF1A1 | PIP4K2C | | | TK2 | | EZR-ROS1 | | PCBP1 |  |  |
| OBSCN | TMSB4X | | | NRGN | | GLUD1 | | OVCH1 |  |  |
| HIST1H4A; HIST1H4F; HIST1H4D; HIST1H4J; HIST2H4A; HIST2H4B; HIST1H4H; HIST1H4C; HIST4H4; HIST1H4E; HIST1H4I; HIST1H4B; HIST1H4K; HIST1H4L | PFN1 | | | MTL5; TESMIN | | ANXA1 | | GPT2 |  |  |
| POTEJ | ACTR2 | | | MYO5A | | FAM20B | | SGSH |  |  |
| PPIA | ANKRD34B | | | SASH1 | | P4HB | | HTRA1 |  |  |
| LDHA | MYOM2 | | | NSD1 | | RFC5 | | TTF1 |  |  |
| ANXA2 | FAR1 | | | ANGPT2 | | PAK3 | | XRCC6 |  |  |
| BCLAF1 | HECTD3 | | | FHOD3 | | SRP68 | | C9orf131 |  |  |
| DST | LRRC42 | | | DOCK5 | | GSTP1 | | FLYWCH1 |  |  |
| SACS | MOB3C | | | CDC5L | | DOCK7 | | TJP1 |  |  |
| SYNE1 | TMEM143 | | | FAM22E; NUTM2E | | SLC25A6 | | ADHFE1 |  |  |
| RPS27A | DIAPH2 | | | ABCC12 | | SARS | | CELF5 |  |  |
| TUBA8 | USP25 | | | SPTBN4 | | DNAJB11 | | GPBP1 |  |  |
| NCL | DNAH6 | | | LPO | | RPS24 | | RRNAD1 |  |  |
| USO1 | MPRIP | | | ERV3-2 | | HNRNPA1 | | TFR2 |  |  |
| SPINK5 | ESRP1 | | | DHX35 | | HNRNPA2B1 | | SEC11C |  |  |
| DNAH17 | SLC35B3 | | | THOC2 | | EIF5A | | XIRP2 |  |  |
| CACNA1E | ZNF76 | | | CCDC157 | | XRCC5 | | CCDC51 |  |  |
| NEB | IGFN1 | | | EYS | | SET | | NAGLU |  |  |
| PPP1R18 | CFLAR | | | FANCC | | SERPINI1 | | DNAH10 |  |  |
| MARK4 | NFRKB | | | BLM | | G3BP2 | | PLD1 |  |  |
| RAPGEF6 | XPC | | | KCNQ5 | | TXN | | TMEM63B |  |  |
| WAPAL; WAPL | SLTM | | | MAPK7 | | PPARD | | TYMS |  |  |
| MUC16 | MYH6 | | | THEMIS | | PMFBP1 | | ETAA1 |  |  |
| APLP2 | PTPN23 | | | ZNF562 | | AKT2 | | HIST1H1D |  |  |
| HSP90B1 | REV1 | | | SETD1A | | RC3H2 | | NOA1 |  |  |
| CEP250 | LINGO4 | | | LINGO3 | | GPALPP1 | | NLRP9 |  |  |
| MYO9B | TRPM6 | | | NAIP | | GOLGA6L2 | | SIPA1 |  |  |
| HMCN1 | FAM20C | | | TBC1D31; WDR67 | | UFSP2 | | HLA-B |  |  |
| ABL1 | TTPAL | | | MUTYH | | FOXP2 | | LILRA6 |  |  |
| ZNF180 | IQGAP3 | | | UTY | | ZC2HC1C | | NISCH |  |  |
| ALMS1 | PPIP5K1 | | | ATP5B | | MBD3 | | PWWP2A |  |  |
| CSE1L | UACA | | | PRPSAP2 | | NIT1 | | C3orf49 |  |  |
| WDR37 | SOX5 | | | TBC1D3B | | ZFP106; ZNF106 | | GUCY1A3 |  |  |
| MLPH | SMS | | | SRPK1 | | SART3 | | BOLA3 |  |  |
| BRIP1 | SYTL5 | | | CRLF2 | | C11orf80 | | ING2 |  |  |
|  |  | | |  | |  | | NTN3 |  |  |
| DNAJC11 | SEMA3A | | | SESN1 | | ARMC8 | | AK8 |  |  |
| CCDC12 | TMEM65 | | | KIAA1217 | | MCM8 | | XRCC5 |  |  |
| COPS5 | ACVR1B | | | PTPN13 | | C10orf67 | | S100A11 |  |  |
| BSCL2 | FNDC3B | | | LOC390937 | | VN1R4 | | DIP2B |  |  |
| MYH4 | ROCK2 | | | RAD18 | | PUS3 | | hCG_2021878 |  |  |
| SPATA21 | TEK | | | ZNF575 | | CEP170B | | SLC6A15 |  |  |
| PRPF40A | SOCS2 | | | HDGFRP3 | | MUC2 | | C4orf22 |  |  |
| CSRP1 | TTC39A | | | PHKB | | SNX8 | | C2orf71 |  |  |
| TMEM115 | TECRL | | | PRO2379 | | ASAP1-IT2; LOC100507117 | | ZNF311 |  |  |
| ANKRD62 | LAMA5 | | | KIAA1841 | | HOXA13 | | DNAJC30 |  |  |
| KIAA1755 | GLI1 | | | CRTAM | | CPPED1 | | WDPCP |  |  |
| FAM135A | KAT2B | | | REEP2 | | ROR2 | | AP4S1 |  |  |
| COL21A1 | ARIH2 | | | KLRC4-KLRK1; KLRK1 | | SERPINB5 | | RPRD1B |  |  |
| NDUFV1 | PAN2 | | | DDX60L | | KHDRBS1 | | VWA5B2 |  |  |
| DDX3X | TACC3 | | | TXNDC2 | | KCNC2 | | RDX |  |  |
| B3GNT4 | NOL10 | | | FAM32A | | FAM129B | | GP5 |  |  |
| USP24 | KIAA0922; TMEM131L | | | USP50 | | NEK9 | | ZNF662 |  |  |
| URB1 | PHF20 | | | COL5A2 | | GTF2B | | ZNF275 |  |  |
| HLA-A | STK38L | | | RS1 | | MAP4K4 | | RBM26 |  |  |
| ARAP2 | CLCA1 | | | TRIM9 | | CLEC4D | | COL17A1 |  |  |
| TPP2 | Uncharacterized protein | | | DMKN | | FAM188B; MINDY4 | | TSPAN5 |  |  |
| FKBP9 | Uncharacterized protein | | | ALLC | | PIBF1 | | ZC2HC1C |  |  |
| DTNA | ADPRHL2 | | | CCDC136 | | RAI1 | | ZNF586 |  |  |
| NEK5 | NVL | | | PYCR1 | | FBXO33 | | DOCK3 |  |  |
| BRCA2 | KCTD8 | | | SYCE3 | | PARP9 | | UQCRFS1P1 |  |  |
| TERF2IP | LRRC6 | | | SIGLEC1 | | TEAD4 | | ZNF333 |  |  |
| IL6R | NFATC2IP | | | CATSPERG | | WAPAL; WAPL | | S100A3 |  |  |
| LOC100294335 | MCF2L2 | | | RCC1 | | TBC1D23 | | CASP8AP2 |  |  |
| HELLS | LATS1 | | | HLA-DRB1 | | PLAGL1 | | MAP1S |  |  |
| TEX33 | ADAMTS17 | | | COMMD9 | | NOX1 | | LOC100506504; PRKCZ-AS1; LOC100294391 |  |  |
| Uncharacterized protein | H1FNT | | | TET1 | | MAGA | | MAGI3 |  |  |
| FBN1 | RGS3 | | | ZNF788 | | ABCC9 | |  |  |  |
| MCOLN1 | TLE6 | | | C3orf67 | | POLR1B | | GLI2 |  |  |
| LOC100996779; LOC283788 | ZNF70 | | | STK32A | | AMPH | | PLA2G7 |  |  |
| GSS | NTNG2 | | | HLA-C | | SPATA16 | | WRN |  |  |
| GLDC | ARMCX4 | | | RIPK1 | | SPATA31C1 | | SPTA1 |  |  |
| SPG7 | TRPC1 | | | GRM1 | | WWP2 | | CD300E |  |  |
| NMRAL1 | ASMT | | |  | |  | |  |  |  |
|  | | |  | | | |  | | | **IFNAR2** |
| DOWNREGULATED GENES | | |  | | | |  | | | **PIK3CB** |
| GAPDH | | SETD2 | | UTRN | IGLON5 | | | ZSCAN5A |  |  |
| TUBA1C | | CEP350 | | FXR1 | ICOSLG | | | GOLGA4 |  |  |
| SPTBN1 | | ARMCX5 | | RNPEP | cDNA FLJ58045 | | | SENP6 |  |  |
| HSPD1 | | RAB7B | | CDK5RAP3 | CNTRL | | | INPP5E |  |  |
| PKM | | ZNF234 | | EIF3F | SELENBP1 | | | EIF2AK4 |  |  |
| HSPA8 | | HPX | | COPZ1 | RANBP3 | | | SLC29A1 |  |  |
| YWHAZ | | TP53BP1 | | NUP85 | MAP1LC3A | | | SHOC2 |  |  |
| MACF1 | | CNGA2 | | TNPO1 | NCAM1 | | | FILIP1 |  |  |
| HIST2H2AA4; HIST2H2AA3 | | KIF2A | | SEC61A1 | TMEM131 | | | PNMA6A; PNMA6C; PNMA6B; PNMA6D |  |  |
| LRBA | | ALDH4A1 | | SNX2 | IL21 | | | PLEKHG4B |  |  |
| CCDC88C | | KIAA1429; VIRMA | | GOSR1 | IQCG | | | ZNF521 |  |  |
| Uncharacterized protein | | RTFDC1 | | GPS1 | LOC100506257 | | | PDE6C |  |  |
| LDHB | | AFF2 | | LANCL2 | SLC17A9 | | | PCBP2 |  |  |
| CHD4 | | ZNF592 | | MOB1B | TAX1BP1 | | | COMMD1 |  |  |
| ACTN4 | | GLB1L3 | | CTSC | ASIC3 | | | ZNF30 |  |  |
| SF3A3 | | AIM1; CRYBG1 | | TRIP13 | ANKRD32; SLF1 | | | CYTB |  |  |
| ZFPM2 | | STOX1 | | CS | LRP1 | | | ZFPM1 |  |  |
| TRIM41 | | NUDCD3 | | HSPA9 | CCDC147; CFAP58 | | | PHACTR1 |  |  |
| NUP214 | | SMARCA5 | | HSD17B4 | TAF5L | | | CNTNAP5 |  |  |
| ATP5A1 | | SVIL | | ANXA7 | Uncharacterized protein | | | IFIT5 |  |  |
| FSCN1 | | ZNF318 | | TXNRD1 | ADCK1 | | | PIEZO1 |  |  |
| NXN | | ANKRD11 | | PSME2 | NEBL | | | KRT72 |  |  |
| MDN1 | | SNRNP70 | | PARP1 | EIF3L | | | NTN4 |  |  |
| PSMD2 | | NAA15 | | DDX6 | LOC100130104 | | | KIF24 |  |  |
| LMNB1 | | EFCAB6 | | TSN | EPB41L3 | | | JMJD1C |  |  |
| HSPA2 | | VIT | | GSTO1 | TRPV6 | | | GABRA4 |  |  |
| NOP56 | | CMYA5 | | NDUFS3 | LOC100127955 | | | RABEPK |  |  |
| HUWE1 | | ARHGEF5 | | CLASP2 | PRSS2 | | | WIZ |  |  |
| ANKHD1-EIF4EBP3; ANKHD1 | | ABCA family | | HNRNPA3 | RBAK | | | C8orf33 |  |  |
| CELF1 | | ZNF888 | | CERS2 | PKD1L1 | | | RPTN |  |  |
| NUBPL | | LAMB3 | | RPS7 | AKAP17A | | | hCG_1648122 |  |  |
| SEC31B | | PPP1R14B | | DNAJB1 | FAM178A; SLF2 | | | PNPLA3 |  |  |
| TTC34 | | GSTM3 | | CAB39 | AARS | | | KLRC4 |  |  |
| HDAC2 | | GDI2 | | ZNF544 | INTS1 | | | SHMT1 |  |  |
| LRRC8A | | C16orf62 | | PDE7B | OAZ1 | | | C9orf172 |  |  |
| FAM73A; MIGA1 | | CEACAM6 | | NR2E1 | TRMT11 | | | OR10J3 |  |  |
| SMU1 | | PIK3CG | | OXR1 | PBRM1 | | | CNGA3 |  |  |
| CD24 | | LRPPRC | | IL23R | CCDC60 | | | PTPRJ |  |  |
| DUOX1 | | KCNK16 | | Uncharacterized protein | CRAT | | | IQCF1 |  |  |
| CRY1 | | KRT34 | | SH3BP1 | ATP13A4 | | | PTER |  |  |
| UNC45B | | CYP8B1 | | FMN1 | SEBOX | | | PRR12 |  |  |
| HMGCS1 | | ATP6V1G2 | | TSPYL5 | SETD8; KMT5A | | | TMEM87A |  |  |
| IDS | | 3 beta HSD | | GTSE1 | KIF19 | | | ALS2CR11; C2CD6 |  |  |
| ELAVL2 | | MICAL3 | | C1orf100 | TTC7A | | | KCNH2 |  |  |
| ARL4C | | HLA-C | | TMEM246 | Gag | | | FLJ43860; MROH5 |  |  |
| PC | | ATP2B2 | | AIM2 | ANKRD13D | | | ULK4 |  |  |
| CORIN | | RPL9P11 | | TBRG4 | VPS37D | | | NOP2 |  |  |
| ZNF177; ZNF559-ZNF177 | | SPAG9 | | SCLT1 | SGSM1 | | | DYNC1H1 |  |  |
| HFM1 | |  | |  |  | | |  |  |  |
