# Supplementary figures and images for: ER stress sensor, glucose regulatory protein 78 (GRP78) regulates redox status in pancreatic cancer thereby maintaining “stemness”
Source: Cell Death Dis. 2019 Feb 12;10(2):132. doi: 10.1038/s41419-019-1408-5 (PMC6372649; doi:10.1038/s41419-019-1408-5)

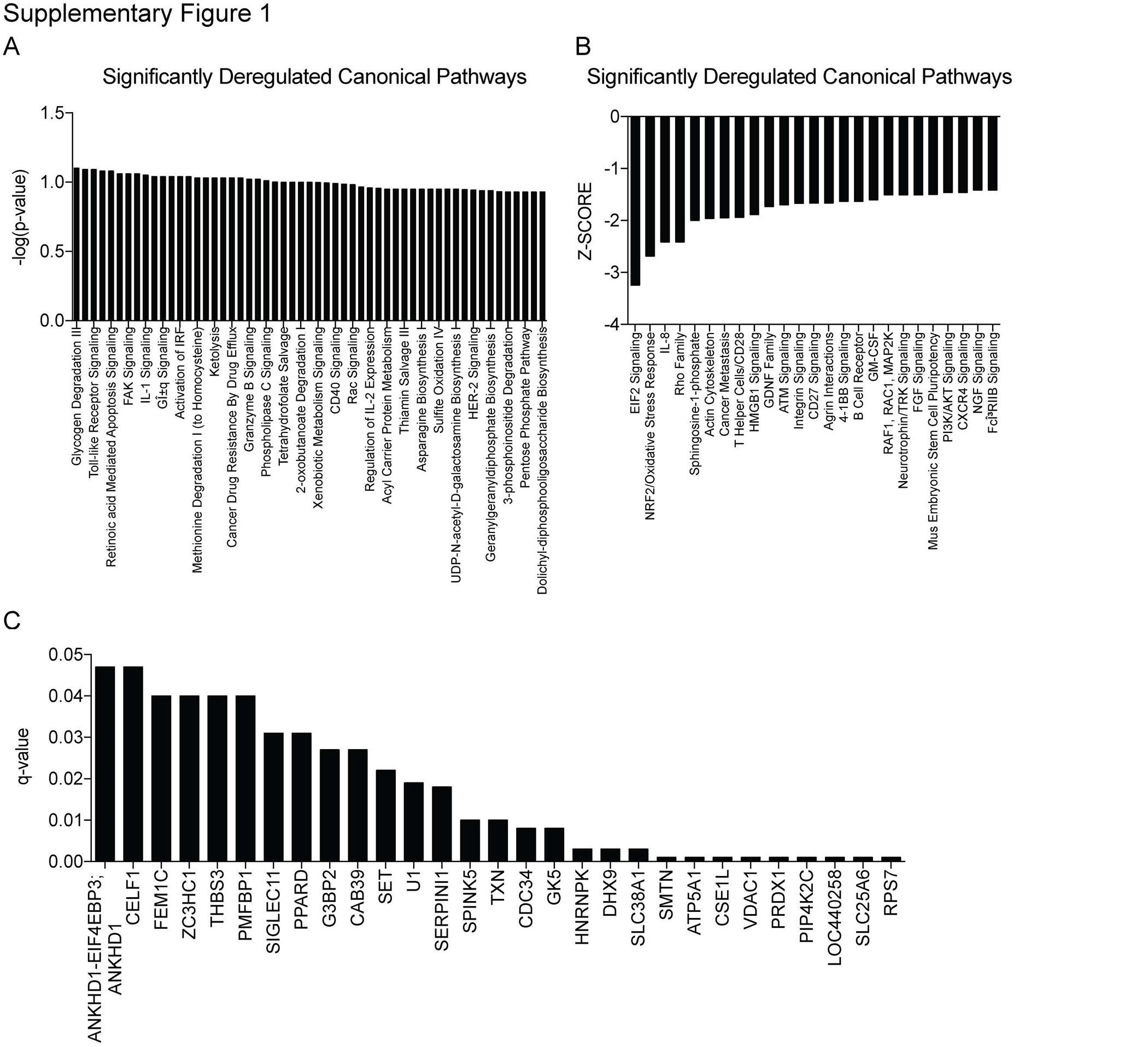

Supplement: Supplementary file 3 — Supplementary Figure 1 [file 41419_2019_1408_MOESM3_ESM.tif]

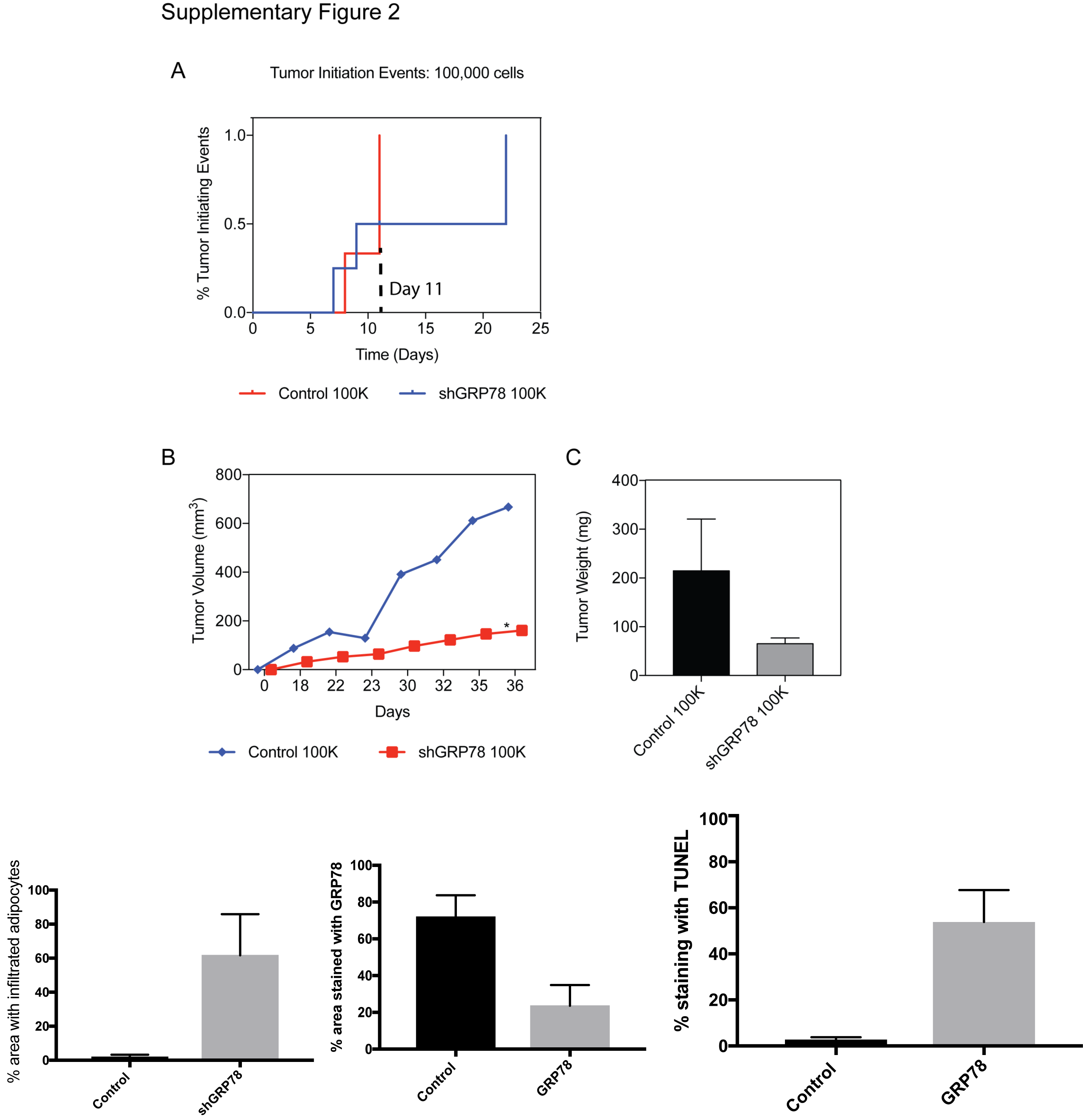

Supplement: Supplementary file 4 — Supplementary Figure 2 [file 41419_2019_1408_MOESM4_ESM.tif]

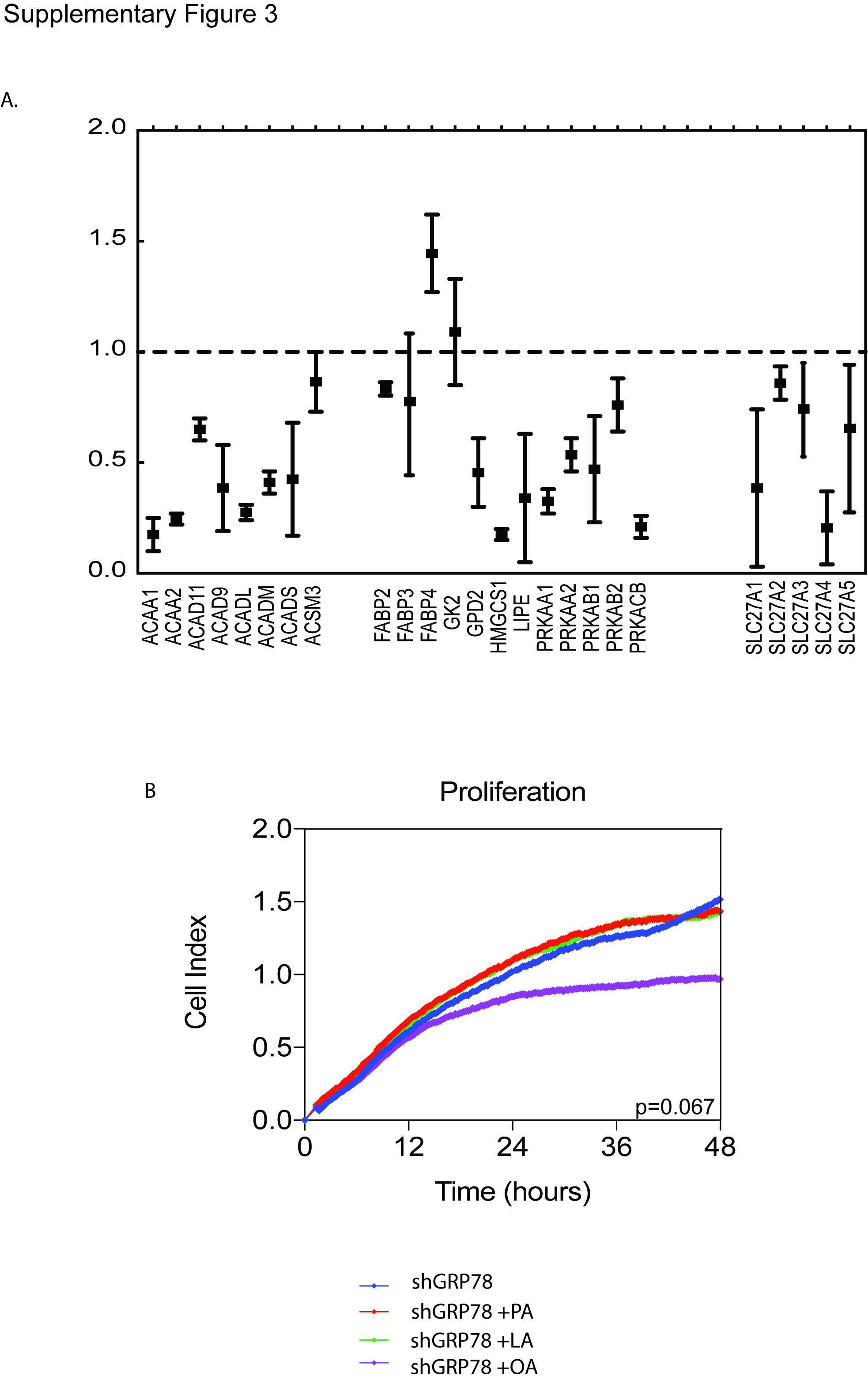

Supplement: Supplementary file 5 — Supplementary Figure 3 [file 41419_2019_1408_MOESM5_ESM.tif]

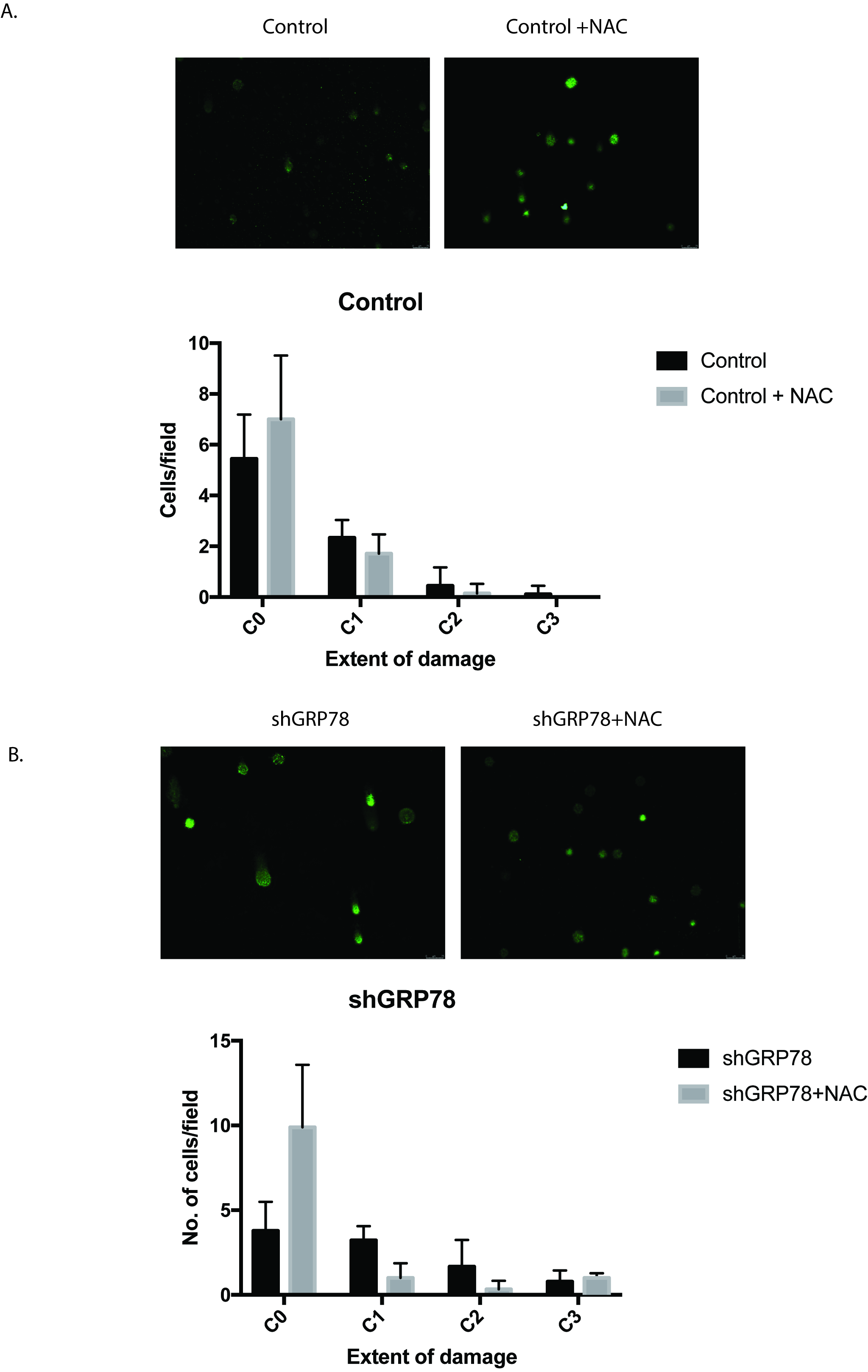

Supplement: Supplementary file 6 — Supplementary Figure 4 [file 41419_2019_1408_MOESM6_ESM.tif]
